# Processing stage flexibility of the SNARC effect: Task relevance or magnitude relevance?

**DOI:** 10.3389/fpsyg.2022.1022999

**Published:** 2022-11-10

**Authors:** Xinrui Xiang, Lizhu Yan, Shimin Fu, Weizhi Nan

**Affiliations:** Department of Psychology and Center for Brain and Cognitive Sciences, School of Education, Guangzhou University, Guangzhou, China

**Keywords:** SNARC effect, flexibility, semantic representation, response selection, two-stage processing model

## Abstract

Previous studies have shown that the processing stage of the spatial-numerical association of response codes (SNARC) effect is flexible. Two recent studies used the same experimental paradigm to check whether the SNARC effect occurred in the semantic-representation stage but reached contradictory conclusions, showing that the SNARC effect was influenced by a magnitude Stroop effect in a magnitude comparison task but not by a parity Stroop effect in a parity judgment task. Those two studies had two distinct operational factors: the task type (magnitude comparison task or parity judgment task, with the numerical magnitude information task-relevant or task-irrelevant) and the semantic representation stage-related interference information (magnitude or parity Stroop effect, with the interference information magnitude-relevant or magnitude-irrelevant). To determine which factor influenced the SNARC effect, in the present study, the Stroop effect was switched in the two tasks based on the previous studies. The findings of four experiments consistently showed that the SNARC effect was not influenced by the parity Stroop effect in the magnitude comparison task but was influenced by the magnitude Stroop effect in the parity judgment task. Combined with the results of those two contradictory studies, the findings indicated that regardless of the task type or the task relevance of numerical magnitude information, magnitude-relevant interference information was the primary factor to affect the SNARC effect. Furthermore, a two-stage processing model that explained the observed flexibility of the SNARC effect was proposed and discussed.

## Introduction

The spatial-numerical association of response codes (SNARC) effect is the most distinctive and robust evidence for spatial-numerical associations, demonstrating that responding to small numbers with the left hand is faster than responding with the right hand, while the converse is true for large numbers (Dehaene et al., 1993). According to the mental number line hypothesis, numbers are represented as a rightward linear organization, with small numbers represented on the left and large numbers on the right. When the representation positions of a number conflict with a participant’s left/right response effector, the SNARC effect is generated (Dehaene et al., 1993; Shaki et al., 2009; Fischer and Shaki, 2014; Basso Moro et al., 2018).

Previous studies have shown that the SNARC effect varies in both the direction and processing stage (Dehaene et al., 1993; van Dijck and Fias, 2011; Fias and van Dijck, 2016; Toomarian et al., 2019; Yan et al., 2022). Particularly in the processing stage, a long-standing debate has been whether the SNARC effect occurs only in the early semantic-representation stage (Mapelli et al., 2003; Fischer et al., 2004; Tlauka, 2010), only in the late response-selection stage (Gevers et al., 2005; Keus and Schwarz, 2005; Daar and Pratt, 2008; Colling et al., 2020; Yan et al., 2021; Pinto et al., 2021a), or in both stages (Basso Moro et al., 2018; Zhang et al., 2020; Nan et al., 2022).

To settle this debate, most behavioral studies have investigated whether the SNARC effect is influenced by manipulating semantic or response-related factors (Hubbard et al., 2005; Tlauka, 2010; Fischer and Shaki, 2014; Basso Moro et al., 2018) based on additive-factor logic. Additive-factor logic is an important method to investigate the stages of information processing. According to this logic, if two experimental factors are interactive, they are thought to occur in the same processing stage; while if they are additive, they are assumed to have different processing stages (Sternberg, 1969; Liu et al., 2010). Recently, two studies followed this logic but have reached contradictory conclusions even when adopting the same approach (Yan et al., 2021; Nan et al., 2022). Nan et al. (2022) simultaneously induced a magnitude Stroop effect (which is semantic-representation related) caused by the compatibility of the target number magnitude information and the background text and a cognitive Simon effect (which is response-selection related) caused by the compatibility of the target number rotation in a magnitude comparison task. They found that the SNARC effect was, respectively, interactive with the magnitude Stroop effect and the Simon effect, indicating that the SNARC effect occurred in both stages. However, Yan et al. (2021) adopted the same experimental paradigm, inducing a parity Stroop effect (caused by the compatibility of the target number parity information and the background text) and a cognitive Simon effect in a parity judgment task and observed that the SNARC effect was interactive with the cognitive Simon effect and additive with the parity Stroop effect, indicating that the SNARC effect occurred only during the response-selection stage.

What caused this inconsistency? There were two different operation factors in the two studies: the task type (a magnitude comparison task vs. a parity judgment task) and the semantic representation stage-related interference information (a magnitude Stroop effect vs. a parity Stroop effect). For the task type, the task relevance of the magnitude information was varied with two tasks (Priftis et al., 2006; Prpic et al., 2016; Deng et al., 2017). In the magnitude comparison task, the magnitude information is task-relevant and actively top-down processed, which could promote the activation of magnitude representation, generating a stable SNARC effect; while in the parity judgment task, the magnitude information is task-irrelevant and automatically bottom-up processed, which could influence the stability of SNARC effect to some extent (Gevers et al., 2006; Georges et al., 2017; Deng et al., 2018). In the case of semantic representation stage-related interference information, the magnitude relevance was changed with two types of interference information. The magnitude Stroop effect is magnitude-relevant because it involves a conflict in the magnitude information processing, while the parity Stroop effect involves a conflict in the parity information processing and is thus magnitude-irrelevant (Hock and Egeth, 1970; MacLeod, 1991).

Previous studies have pointed out that the representation of magnitude information plays a necessary role in the SNARC effect (Walsh, 2003; Shaki and Fischer, 2018; Pinto et al., 2019b; Prpic et al., 2021). The task relevance of magnitude information and the magnitude relevance of interference information may both influence the magnitude representation, thus showing the flexibility of the SNARC effect in the studies of Yan et al. (2021) and Nan et al. (2022). Nevertheless, it is unclear which of these two factors is the key to affect the SNARC effect.

Therefore, based on the experimental paradigm of Yan et al. (2021) and Nan et al. (2022), we changed the type of Stroop effect in the magnitude comparison task and parity judgment task to determine which factor produced the contradictory findings. In Experiment 1, a parity Stroop effect was induced in a magnitude comparison task. In Experiment 2, a magnitude Stroop effect was induced in a parity judgment task. In particular, we were interested in verifying whether switching the type of Stroop effect (the semantic representation stage-related interference information) in the same task type would influence the SNARC effect. Namely, if the task relevance of magnitude information is the primary factor, the SNARC effect would be affected by the semantic representation stage-related interference information when the magnitude information is task-relevant, regardless of the type of interference information. This would be shown that the SNARC effect would be influenced by the parity Stroop effect in the magnitude comparison task but not by the magnitude Stroop effect in the parity judgment task, repeating the previous results (Yan et al., 2021; Nan et al., 2022). In contrast, if the magnitude relevance of interference information is the primary factor, the SNARC effect would only be influenced by the semantic representation stage-related interference information that is magnitude-relevant, regardless of the task type. This would be shown that the SNARC effect would be affected by the magnitude Stroop effect but not by the parity Stroop effect.

In addition, both Yan et al. (2021) and Nan et al. (2022) induced the cognitive Simon effect and found that it affected the SNARC effect. The Simon effect is usually classified into two types: the visuomotor Simon effect and the cognitive Simon effect (Wascher et al., 2001; Wiegand and Wascher, 2005, 2007). The visuomotor Simon effect is caused by the exogenous location information of the stimulus, which decreases along RTs since the decay of the visual spatial information. The cognitive Simon effect is caused by the mutual interference between the cognitive coding of the stimulus location and the reaction location, which remains stable or strengthens at longer RTs because the stimulus feature translation into the spatial representation requires more time (Toomarian et al., 2019; Yan et al., 2021). Thus, the interference caused by the cognitive Simon effect on the spatial representation of the SNARC effect is more stable. In our study, the cognitive Simon effect was also induced, and we speculated that the cognitive Simon effect would consistently affect the SNARC effect in both tasks.

## Methods and results

### Experiment 1a

In this experiment, we aimed to assess the influence on the SNARC effect when switching the semantic representation stage-related interference information to magnitude-irrelevant under the condition that magnitude information was task-relevant. Thus, a parity Stroop effect was induced in a magnitude comparison task. The interference information induced by the background text (Chinese character: “奇” or “偶,” meaning “odd” and “even,” respectively) referred to the parity of numbers and was thus magnitude-irrelevant. If the task relevance of magnitude information is the primary factor, the results would show an interaction between the SNARC and parity Stroop effects. If the magnitude relevance of interference information is the primary factor, the results would show no interaction between the SNARC and parity Stroop effects.

#### Participants

The sample sizes of all the experiments in the present study were estimated by G*power 3.1 (Faul et al., 2007). With a strict statistical test force (1 − *β*) of 0.95 for detecting an effect at an alpha level of 0.05, a minimum of 23 participants were required to achieve an actual power of.95. According to this calculation and the possibility of participant loss (unusual data or technical issues), the actual sample size (approximately 30) slightly exceeded the estimated sample size (23) to reach the actual power.

Thirty-five undergraduate students (18–24 years old, average age: 19.8 years old; 10 men) from Guangzhou University participated in Experiment 1a. All the participants reported that they had no history of neurological or psychiatric issues; all participants were right-handed and had a normal or corrected-to-normal vision. Each participant voluntarily enrolled and signed an informed consent form before the experiments. This study was approved by the Institutional Review Board of the Educational School, Guangzhou University.

#### Apparatus and stimuli

**Figure 1:**
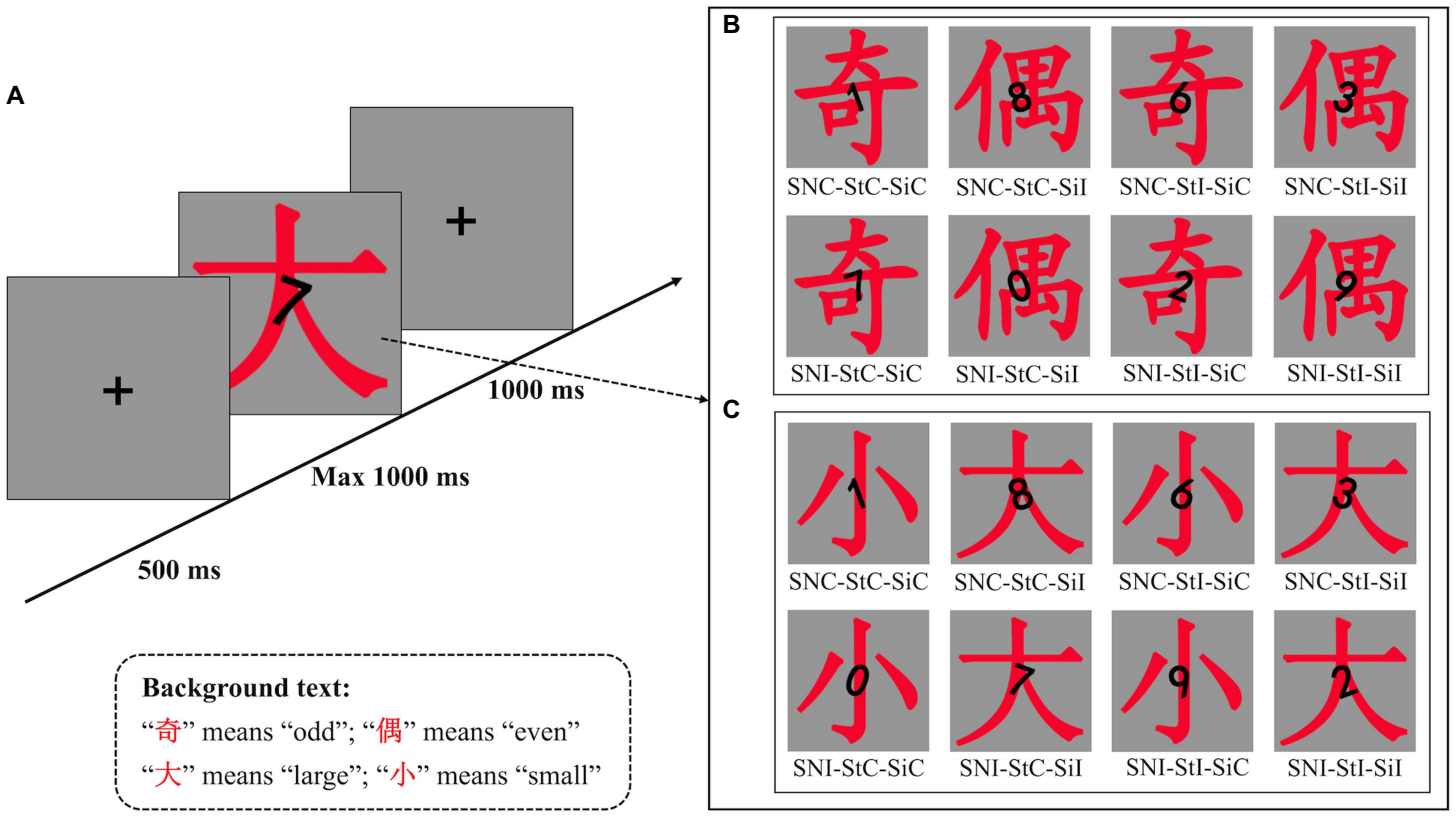
**(A)** Schematic illustration of the task. At the start of the experiment, a fixation cross was displayed for 500 ms, followed by a target number displayed for 1,000 ms; participants were instructed to compare the target numbers with the numbers “4” and “5” in Experiments 1a and 1b and to judge the parity of the target number in Experiments 2a and 2b. After the target number disappeared, a fixation cross was presented at the center of the screen for 1,000 ms. **(B)** The stimuli of Experiment 1a. Conditions for the combinations of the SNARC, parity Stroop, and cognitive Simon effects. There are eight conditions in total, each containing three effect attributes. The naming rules for the eight conditions are as follows: “SN” stands for the SNARC effect, “St” stands for the parity Stroop effect, “Si” stands for the cognitive Simon effect, “C” stands for congruent, and “I” stands for incongruent. For example, “SNC-StC-Sic” means SNARC congruent, parity Stroop congruent and cognitive Simon congruent. The other names have similar meanings. For the left-hand response to small numbers, the SNARC effect is congruent. For the cognitive Simon effect, the number 1 titled to the left is congruent, while the number 1 titled to the right is incongruent. The stimuli of Experiment 1b were the same as those of Experiment 1a, except that the target numbers included only four numbers (1, 2, 7, and 8). **(C)** The stimuli of Experiment 2a. Similar to Experiment 1a, the conditions for the combinations of the SNARC, cognitive Simon and magnitude Stroop effects. For the left-hand response to odd numbers, for the SNARC effect, the number 1 is congruent, while the number 7 is not. For the cognitive Simon effect, the number 1 titled to the left is congruent, while the number 1 titled to the right is not. For the magnitude Stroop effect, if the background text is “小” (meaning “small”), then the number 1 is congruent and the number 7 is not.

All participants were seated in a sound-attenuated and dimly lit chamber approximately 60 cm away from a 27-in. liquid-crystal display (LCD) monitor (resolution: 1,024 × 768 pixels, vertical refresh rate: 100 Hz), with their eyes fixed with the center of the monitor. All stimuli were presented on a gray panel (300 × 300 pixels) with a black background and were composed of the numbers (0, 1, 2, 3, 6, 7, 8, or 9; size 1 × 1.5° in boldface), number rotation (clockwise rotation with +20° or-20°, with 0° as 12 o’clock) and background text (Chinese character: “奇” or “偶,” meaning “odd” and “even,” respectively; size 5 × 6° in regular script). The stimuli of the number and background text were presented in the center of the screen, with the number overlaid on the background text (Figure 1B). The stimulus presentation and manual response measurements were controlled by E-Prime 2.0 software (Psychological Software Tools, Inc., Pittsburgh, PA).

#### Design and procedures

A 2 (SNARC effect: congruent and incongruent) × 2 (parity Stroop effect: congruent and incongruent) × 2 (cognitive Simon effect: congruent and incongruent) within-subject design was used. The SNARC effect was manipulated by the response rule (congruent: small/large numbers with left/right-hand response, incongruent: small/large numbers with right/left-hand response; Dehaene et al., 1993); the parity Stroop effect was manipulated by the number parity and the background text (congruent: odd/even numbers with “奇”/“偶” character, incongruent: odd/even numbers with “偶”/“奇” character; MacLeod, 1991; Szucs and Soltesz, 2007); and the cognitive Simon effect was manipulated by the number rotation and response hand (congruent: number rotation of −20°/+20° with left/right-hand response; incongruent: number rotation of −20°/+20° with right/left-hand response; Wang et al., 2014; Yan et al., 2021). The combination of these factors resulted in eight different conditions across all trials (Figure 1B). In the formal experiment, the 320 trials were divided into 8 blocks with 40 trials for each condition. Half of the blocks were used for the SNARC congruent response rule (small/large numbers with left/right-hand response), while the other half were used for the SNARC incongruent response rule (small/large numbers with right/left-hand response). The order of the response rules was counterbalanced between subjects.

Participants were instructed to focus on the central fixation during the whole experiment and to estimate the number as quickly and accurately as possible while ignoring the rotation of the number and the background text by pressing a button on a keyboard (the “S” button for smaller than “4” and “5” numbers with the left index finger and the “L” button for larger than “4” and “5” numbers with the right index finger in the SNARC congruent condition; the stimulus–response rule was reversed in the SNARC incongruent condition).

Before the formal experiment blocks, participants would complete five training blocks. In the first training block, they were asked to identify the numbers (0, 1, 2, 3, 6, 7, 8, or 9) across 16 trials to become acquainted with the response rule. In the second training block, which consisted of 40 trials with the letters A and B as stimuli (clockwise rotation of −20°/+20°), participants were instructed to identify the rotation of the stimuli to reinforce the connection between the stimulus rotation and the response hand for the cognitive Simon effect. The third training block consisted of 40 trials with the numbers 3 and 6 as stimuli (clockwise rotation of −20°/+20°) to test the cognitive Simon effect. If there was no cognitive Simon effect, the program would return to the second practice test. In the fourth training block, which consisted of 120 trials, participants were instructed to determine the parity of the numbers (0, 1, 2, 3, 6, 7, 8, or 9) and Chinese characters (“奇” or “偶”) to strengthen the connection between the numbers and Chinese characters for the parity Stroop effect. The fifth training block was identical to the formal experiment blocks except that it only included 10 trials to familiarize the participants with the formal experiment.

As shown in Figure 1A, a fixation cross (size: 0.7 × 0.7°) was presented at the beginning of each trial. After 500 ms, a target with a black number (clockwise rotation of −20°/+20°) on a red Chinese character appeared for either 1,000 ms or until there was a response. Then, a fixation cross appeared at the center of the screen for 1,000 ms.

#### Statistical analysis

The error rates (ER) and reaction times (RT) for correct responses were recorded for analysis. The data of 3 of the 35 participants were excluded from the analysis. One participant had an error rate greater than 15%. One participant had previously participated in a similar experiment and guessed the purpose of the experiment, and another failed to comply with the instruction during the experiment.

The mean accuracy of the remaining participants was 97.7%. The trials with errors, RTs shorter than 200 ms, and RTs greater than 3 standard deviations (6.7%) in each condition were excluded from the RT analysis. The following RT and ER results were obtained using repeated-measures analysis of variance (ANOVA) with the SNARC effect (congruent, incongruent), parity Stroop effect (congruent, incongruent), and cognitive Simon effect (congruent, incongruent) as within-subjects factors. The significance level was set at *α* < 0.05 for all ANOVAs. Bonferroni corrections were used for pairwise comparisons. To highlight the interaction between the SNARC effect and the other two effects, the interaction was mainly analyzed by estimating whether the effect size of the SNARC effect changed with the congruent and incongruent conditions of the other two effects.

Besides, the relationship between the RTs and ERs was analyzed to determine whether there was a trade-off or correlation. If there are trade-offs or correlations between the RT and ER, the inverse efficiency scores (IES), equal to the mean RT divided by the proportion of correct responses (expressed in ms), would be introduced as a new ANOVA indicator; the IES is usually used to discount possible criterion shifts or speed-accuracy trade-offs (Townsend and Ashby, 1983; Bruyer and Brysbaert, 2011).

In addition, the Bayes factors (BF_10_) were calculated for theoretically meaningful null interactions (Weidler et al., 2022). A BF_10_ less than 1/3 provides substantial evidence for the null while a BF_10_ larger than 3 provides substantial evidence for the alternative, and any BF_10_ between 1/3 and 1 indicates that the evidence is too weak to reject or accept the null interaction (Wagenmakers et al., 2011).

#### Results

**Figure 2:**
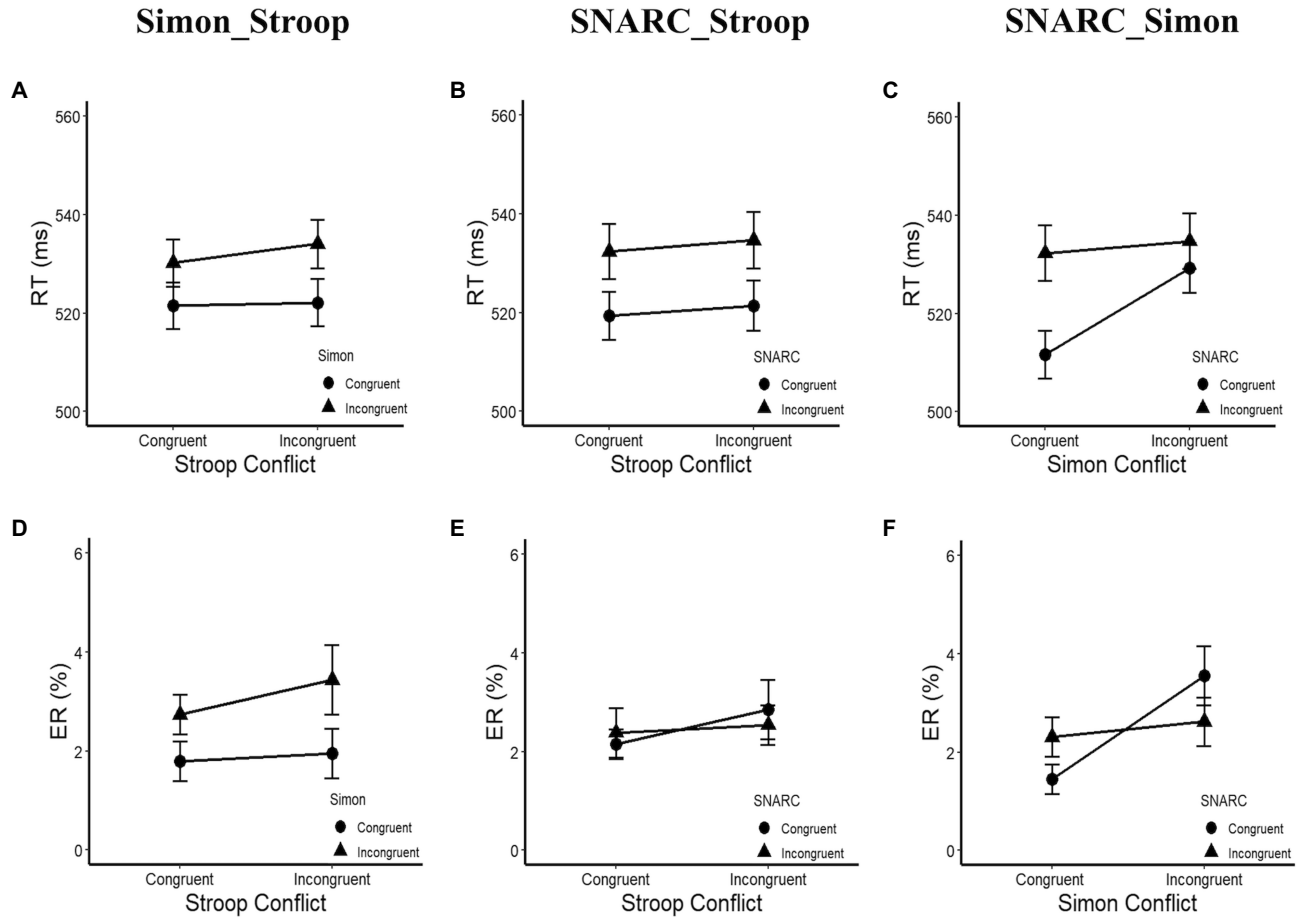
RT and ER in Experiment 1a. **(A,D)** Present the interaction between the parity Stroop effect and the cognitive Simon effect. The circle symbol represents the Simon congruent condition, the triangle symbol represents the Simon incongruent condition. **(B,E)** Depict the interaction between the SNARC effect and the parity Stroop effect. The circle symbol represents the SNARC congruent condition, the triangle symbol represents the SNARC incongruent condition. **(C,F)** Illustrate the interaction between the SNARC effect and the cognitive Simon effect. The representation of circle and triangle symbols is the same as the **(B,E)**. The error bar represents the standard error.

In terms of the RTs (Figures 2A–C; Table 1), a main effect of the cognitive Simon effect was observed, with *F*(1, 31) = 11.32, *p* = 0.002, *η_p_*^2^ = 0.27, and a longer RT in the Simon incongruent condition (532 ± 10 ms) than in the Simon congruent condition (522 ± 9 ms). An interaction between the SNARC effect and the cognitive Simon effect was observed, with *F*(1, 31) = 13.84, *p* = 0.001, and *η_p_*^2^ = 0.31. A simple effect analysis showed that the effect size of the SNARC effect in the Simon congruent condition (21 ± 9 ms) was larger than that in the Simon incongruent condition (5 ± 10 ms), *t*(32) = 3.72, *p* = 0.001, *d* = 0.66. Besides, the interaction between the SNARC effect and the parity Stroop effect was not observed, *F*(1, 31) = 0.003, *p* = 0.958, *η_p_*^2^ < 0.01, BF_10_ = 0.18. Also, the interaction between the cognitive Simon effect and the parity Stroop effect was not observed, *F*(1, 31) = 0.72, *p* = 0.403, *η_p_*^2^ = 0.02, BF_10_ = 0.21. No other significant main effects or interactions were observed, with all *p* values greater than 0.05.

**Table 1:** The mean and standard error of RTs and ERs in Experiment 1a.

		C/C	C/I	I/C	I/I
Stroop/Simon	RT (ms)	522 (10)	530 (9)	522 (9)	534 (10)
	ER (%)	1.8 (0.4)	2.7 (0.5)	2.0 (0.4)	3.4 (0.7)
SNARC/Simon	RT (ms)	512 (10)	529 (10)	532 (11)	535 (11)
	ER (%)	1.4 (0.3)	3.6 (0.6)	2.3 (0.4)	2.6 (0.5)
SNARC/Stroop	RT (ms)	519 (10)	522 (10)	532 (11)	535 (11)
	ER (%)	2.1 (0.3)	2.9 (0.6)	2.4 (0.5)	2.5 (0.4)

C/C means that the two effects are both in the congruent condition, while I/C means that the former effect is in incongruent condition while the latter is congruent condition.

In terms of the ERs (Figures 2D–F; Table 1), a main effect of the cognitive Simon effect was observed, with *F*(1, 31) = 12.76, *p* = 0.001, *η_p_*^2^ = 0.29, and a larger ER in the Simon incongruent condition (3.1 ± 0.5%) than in the Simon congruent condition (1.9 ± 0.3%). An interaction between the SNARC effect and the cognitive Simon effect was observed, with *F*(1, 31) = 10.57, *p* = 0.003, and *η_p_*^2^ = 0.25. A simple effect analysis showed that the effect size of the SNARC effect in the Simon congruent condition (0.9 ± 0.4%) was larger than that in the Simon incongruent condition (−0.9 ± 0.4%), *t*(32) = 3.25, *p* = 0.003, *d* = 0.57. Besides, the interaction between the SNARC effect and the parity Stroop effect was not observed, *F*(1, 31) = 0.67, *p* = 0.418, *η_p_*^2^ = 0.02, BF_10_ = 0.28. Also, the interaction between the cognitive Simon effect and the parity Stroop effect was not observed, *F*(1, 31) = 0.44, *p* = 0.003, *η_p_*^2^ = 0.25, BF_10_ = 0.25. No other significant main effects or interactions were observed, with all *p* values greater than 0.05.

In addition, the Pearson correlation coefficient of RT and ER was nonsignificant, with *r* = −0.068 and *p* = 0.279, indicating that there was no trade-off or correlation between the two. Thus, there was no need to introduce the IES for further analysis.

#### Discussion

The results showed that there was no interaction between the cognitive Simon effect and the parity Stroop effect, verifying that these two factors are certainly processed in different stages (Li et al., 2014). The interactive combination of the SNARC and cognitive Simon effects showed that the SNARC effect is affected by the response-selection stage-related interference information, which is consistent with the results of previous studies (Gevers et al., 2005; Yan et al., 2021; Nan et al., 2022). Moreover, the additive combination of the SNARC and parity Stroop effects was observed, indicating that the SNARC effect is not affected when the interference information is magnitude-irrelevant and the magnitude information is task-relevant. But, Nan et al. (2022) observed the interactive combination of the SNARC and the magnitude Stroop effects in a magnitude comparison task. Combined with these results, it could be found that the SNARC effect was modulated by manipulating the magnitude relevance of interference information when the magnitude information was task-relevant. This finding supports the hypothesis that the magnitude relevance of interference information is the primary factor affecting the SNARC effect.

### Experiment 1 b

In Experiment 1a, we used the numbers 0–9 (except 4 and 5) to be consistent with most previous studies (Nuerk et al., 2005; Schwarz and Muller, 2006; Pressigout et al., 2019). However, some studies have also simplified the experiment by adopting four target numbers (e.g., 1, 2, 7, and 8; Ninaus et al., 2017; Shaki and Fischer, 2018; Yan et al., 2021). Thus, in Experiment 1b, we adopted four target numbers (1, 2, 7, and 8) to determine whether the main results of Experiment 1a were robust and repeatable.

#### Participants

Thirty undergraduate students (18–23 years old, average age: 20.0 years old; 13 men) from Guangzhou University participated in Experiment 1b. All the participants reported that they had no history of neurological or psychiatric issues; all participants were right-handed and had normal or corrected-to-normal vision.

#### Apparatus, stimuli, and design

Experiment 1b was identical to Experiment 1a, except that the target number range was changed to four numbers (1, 2, 7, and 8).

#### Statistical analysis

The analysis procedure of Experiment 1b was the same as that of Experiment 1a. The data of all participants were used for analysis, with a mean accuracy of 97.7%. The trials with errors, RTs shorter than 200 ms, or RTs greater than 3 standard deviations (6.2%) in each condition were excluded from the RT analysis.

#### Results

**Figure 3:**
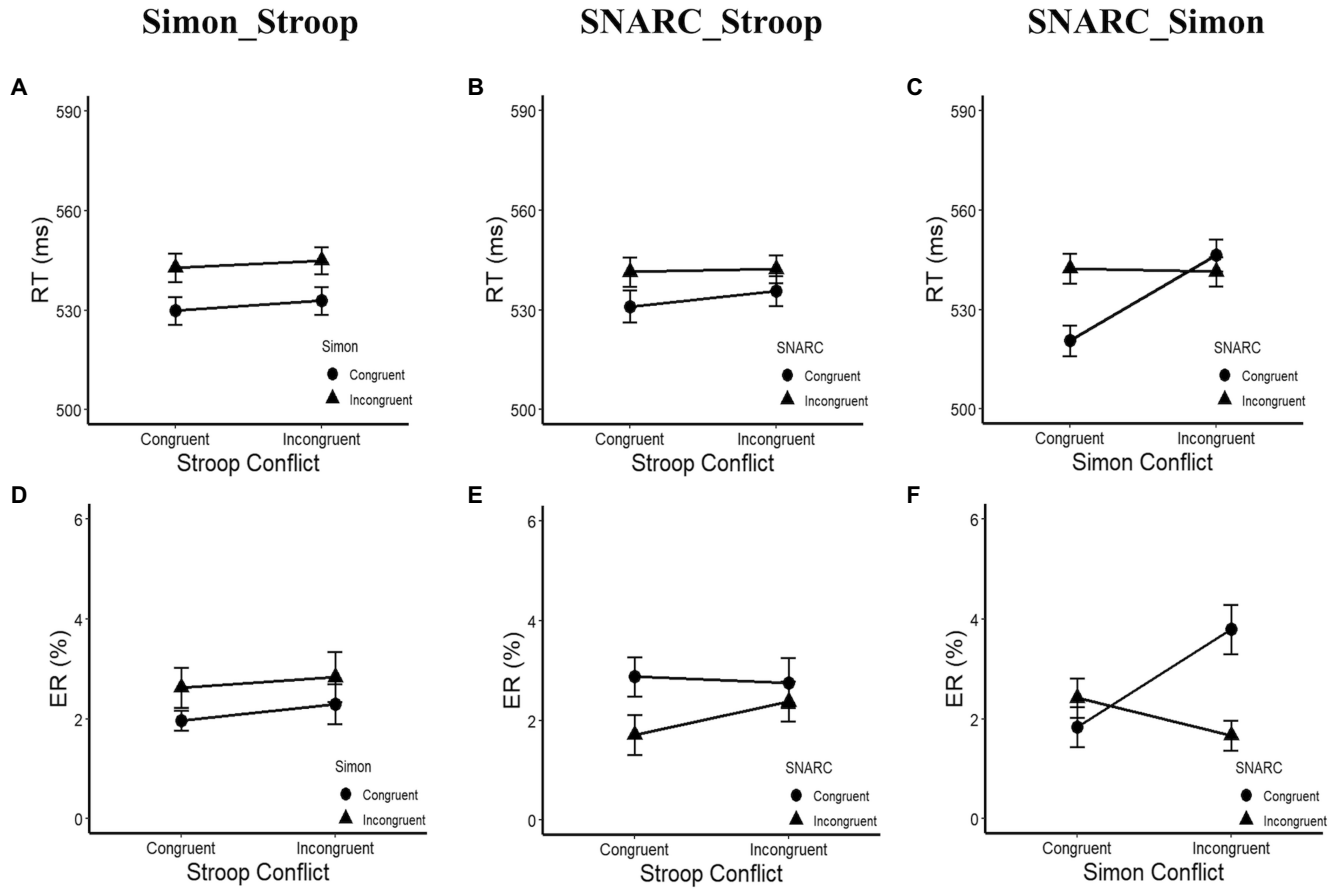
RT and ER in Experiment 1b. **(A,D)** Present the interaction between the parity Stroop effect and the cognitive Simon effect. The circle symbol represents the Simon congruent condition, the triangle symbol represents the Simon incongruent condition. **(B,E)** Depict the interaction between the SNARC effect and the parity Stroop effect. The circle symbol represents the SNARC congruent condition, the triangle symbol represents the SNARC incongruent condition. **(C,F)** Illustrate the interaction between the SNARC effect and the cognitive Simon effect. The representation of circle and triangle symbols is the same as the **(B,E)**. The error bar represents the standard error.

In terms of the RTs (Figures 3A–C; Table 2), a main effect of the cognitive Simon effect was observed, with *F*(1, 29) = 17.42, *p* < 0.001, *η_p_*^2^ = 0.38, and a longer RT in the Simon incongruent condition (544 ± 8 ms) than in the Simon congruent condition (531 ± 8 ms). An interaction between the SNARC and cognitive Simon effects was observed, with *F*(1, 29) = 36.78, *p* < 0.001, and *η_p_*^2^ = 0.56. A simple effect analysis showed that the effect size of the SNARC effect in the Simon congruent condition (22 ± 8 ms) was larger than that in the Simon incongruent condition (−5 ± 7 ms), *t*(30) = 6.06, *p* < 0.001, *d =* 1.11. Besides, the null interaction between the SNARC effect and the parity Stroop effect was observed, *F*(1, 29) = 2.14, *p* = 0.154, *η*_p_^2^ = 0.07, BF_10_ = 0.24. Also, the null interaction between the cognitive Simon effect and the parity Stroop effect was observed, *F*(1, 29) = 0.07, *p* = 0.800, *η_p_*^2^ < 0.01, BF_10_ = 0.19. No other significant main effects or interactions were observed, with all *p* values greater than 0.05.

**Table 2:** The mean and standard error RTs and ERs in Experiment 1b.

		C/C	C/I	I/C	I/I
Stroop/Simon	RT (ms)	530 (9)	543 (9)	533 (8)	545 (8)
	ER (%)	2.0 (0.5)	2.6 (0.4)	2.3 (0.4)	2.8 (0.5)
SNARC/Simon	RT (ms)	521 (9)	546 (10)	542 (9)	541 (9)
	ER (%)	1.8 (0.4)	3.8 (0.5)	2.4 (0.4)	1.7 (0.3)
SNARC/Stroop	RT (ms)	531 (10)	536 (9)	541 (9)	542 (9)
	ER (%)	2.9 (0.4)	2.8 (0.5)	1.7 (0.4)	2.4 (0.4)

C/C means that the two effects are both in the congruent condition, while I/C means that the former effect is in incongruent condition while the latter is congruent condition.

In terms of the ERs (Figures 3D–F; Table 2), a main effect of the SNARC effect was observed, with *F*(1, 29) = 4.28, *p* = 0.048, *η_p_*^2^ = 0.13, and a larger ER in the SNARC congruent condition (2.8 ± 0.4%) than in the SNARC incongruent condition (2.0 ± 0.3%). An interaction between the SNARC and cognitive Simon effects was observed, with *F*(1, 29) = 22.42, *p* < 0.001, and *η_p_*^2^ = 0.44. A simple effect analysis showed that the effect size of the SNARC effect in the Simon incongruent condition (−2.1 ± 0.5%) was larger than that in the Simon congruent condition (0.6 ± 0.5%), *t*(30) = 4.74, *p* < 0.001, *d* = 0.86. Besides, the null interaction between the SNARC effect and the parity Stroop effect was observed, *F*(1, 29) = 1.62, *p* = 0.213, *η*_p_^2^ = 0.05, BF_10_ = 0.34. Also, the null interaction between the cognitive Simon effect and the parity Stroop effect was observed, *F*(1, 29) = 0.03, *p* = 0.861, *η*_p_^2^ < 0.01, BF_10_ = 0.19. No other significant main effects or interactions were observed, with all *p* values greater than 0.05.

In addition, the Pearson correlation coefficient of RT and ER was nonsignificant, with *r* = 0.014 and *p* = 0.830, indicating that there was no trade-off or correlation between the two. Thus, there was no need to introduce the IES for further analysis.

#### Discussion

The results of Experiment 1b replicated the findings of Experiment 1a: an interaction between the SNARC and cognitive Simon effects was observed, as well as the absence of interactions between the parity Stroop and cognitive Simon effects and the SNARC and parity Stroop effects. These results showed that the SNARC effect is certainly not affected by magnitude-irrelevant interference information when the magnitude information is task-relevant, implying that the primary factor influencing the SNARC effect might be the magnitude relevance of interference information.

### Experiment 2a

In Experiment 2a, we tried to investigate the influence of the magnitude relevance of interference information on the SNARC effect when the magnitude information was task-irrelevant, to jointly confirm the primary factors affecting the SNARC effect with Experiment 1. For this reason, a magnitude Stroop effect was induced in a parity judgment task. The interference information introduced by the background text (Chinese character: “大” or “小,” meaning “large” and “small,” respectively) referred to the numerical magnitude and was thus magnitude-relevant. If the task relevance of magnitude information is the primary factor, an interaction between the SNARC effect and the magnitude Stroop effect should be observed. Conversely, if the magnitude relevance of interference information is the primary factor, a null interaction between the SNARC and magnitude Stroop effects should be observed.

#### Participants

Thirty-three undergraduate students (18–25 years old, average age: 20.4 years old; 8 men) from Guangzhou University participated in Experiment 2a. All the participants reported that they had no history of neurological or psychiatric issues; all participants were right-handed and had normal or corrected-to-normal vision.

#### Apparatus, stimuli, design, and procedure

The apparatus and procedure were the same as in Experiment 1a, but the stimuli and design had some changes in Experiment 2a. The background text of the stimulus was changed from “奇”/“偶” (meaning “odd” and “even”) to “大”/“小” (meaning “large” and “small”) to transform the parity Stroop effect into the magnitude Stroop effect (Figure 1C). In terms of the design, the magnitude Stroop effect was manipulated by the numerical magnitude and the background text (congruent: large/small number with “大”/“小” character; incongruent: small/large number with “大”/“小” character).

In Experiment 2a, the formal blocks consisted of 160 trials, which were divided into 4 blocks with 20 trials for each condition. The participants were instructed to press a button to indicate the parity of the target number (the “S” button with the left index finger for even numbers and the “L” button with the right index finger for odd numbers; the response map was counterbalanced between the subjects) as quickly and accurately as possible while ignoring the rotation of the number and the background text.

Before the formal blocks, the participants completed four training blocks. The first training block consisted of 40 trials with the letters A and B as stimuli (clockwise rotation of −20°/+20°) to strengthen the connection between the rotation of the stimulus and the response hand for the cognitive Simon effect. The second training block, which consisted of 40 trials, used the Chinese characters “大” and “小” (meaning large and small) as stimuli to strengthen the connection between the numerical magnitude and the Chinese characters for the magnitude Stroop effect. The third training block consisted of 16 trials; the participants were asked to identify the parity of the numbers (0–9, except references 4 and 5) to familiarize themselves with the response rule. The fourth training block consisted of 40 trials and was the same as the formal experiment to familiarize the participants with the formal blocks and to test the magnitude Stroop effect and cognitive Simon effect. If there were no cognitive Simon and magnitude Stroop effects, the program would return to the first training block.

The procedure of the formal blocks was the same as that of Experiment 1a (Figure 1A).

#### Statistical analysis

The analysis procedure of Experiment 1a was used for Experiment 2a. One participant was excluded from the analysis since the error rate was greater than 15%. The mean accuracy of the remaining participants was 95.1%. The trials with errors, RTs shorter than 200 ms, or RTs greater than 3 standard deviations (10.7%) in each condition were excluded from the RT analysis.

#### Results

**Figure 4:**
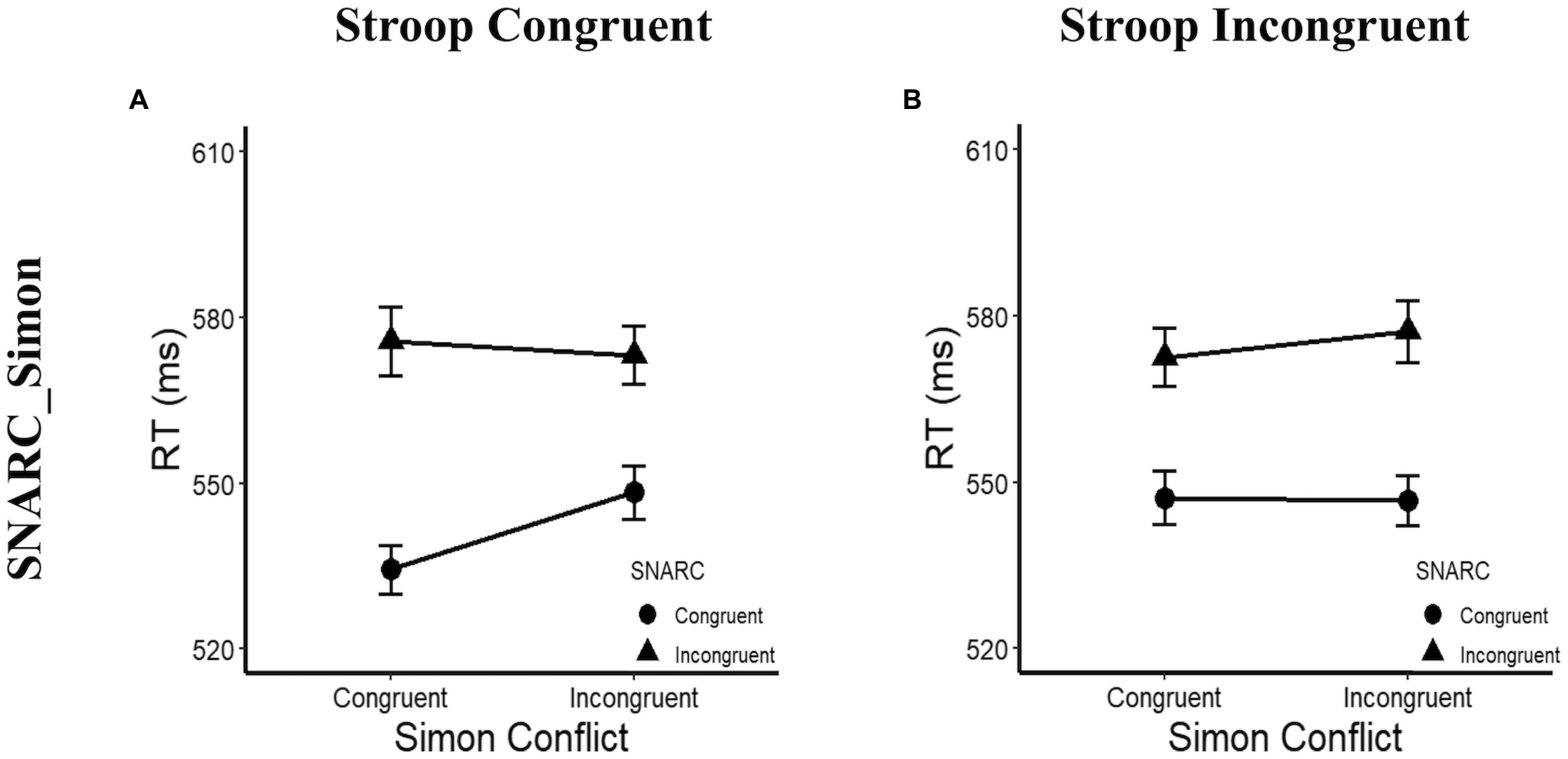
RTs in the *post hoc* multiple comparison of Experiment 2a. **(A)** Illustrate the interaction between the SNARC and cognitive Simon effects in the Stroop congruent condition. The circle symbol represents the SNARC congruent condition, the triangle symbol represents the SNARC incongruent condition. **(B)** Present the interaction between the SNARC and cognitive Simon effects in the Stroop incongruent condition. The representation of circle and triangle symbols is the same as the **(A)**. The error bar represents the standard error.

In terms of the RTs, a main effect of the SNARC effect was observed, with *F*(1, 31) = 34.28, *p* < 0.001, *η_p_*^2^ = 0.53, and a longer RT in the SNARC incongruent condition (575 ± 11 ms) than in the SNARC congruent condition (544 ± 9 ms). A three-way interaction among the SNARC effect, magnitude Stroop effect and cognitive Simon effect was observed, with *F*(1, 31) = 4.53, *p* = 0.041, and *η_p_*^2^ = 0.13. A *post hoc* multiple comparison test showed that in the magnitude Stroop congruent condition, there was a marginally significant interaction between the SNARC and cognitive Simon effects, with *F*(1, 31) = 3.58, *p* = 0.068, and *η_p_*^2^ = 0.10, showing that the effect size of the SNARC effect in the Simon congruent condition (41 ± 8 ms) tended to be larger than that in the Simon incongruent condition (25 ± 7 ms); however, in the magnitude Stroop incongruent condition, there was no interaction between the SNARC and cognitive Simon effects, with *F*(1, 31) = 0.71, *p* = 0.407, and *η_p_*^2^ = 0.02 (Figures 4A,B; Table 3). Besides, the interaction between the cognitive Simon effect and the magnitude Stroop effect was not observed, *F*(1, 31) = 0.48, *p* = 0.493, *η_p_*^2^ = 0.02, BF_10_ = 0.23. No other significant main effects or interactions were observed, with all *p* values greater than 0.05.

**Table 3:** *Post hoc* multiple comparison results of Experiment 2a for RTs.

			C/C	C/I	I/C	I/I
SNARC/Simon	Stroop congruent	RT (ms)	534 (9)	548 (10)	576 (13)	573 (11)
	Stroop incongruent	RT (ms)	547 (9)	547 (9)	573 (10)	577 (11)

C/C means that the SNARC effect and the Simon effect are both in the congruent condition, while I/C means that the SNARC effect is in the incongruent condition and the Simon effect is in the congruent condition. Stroop Congruent means that the interaction of two effects was in Stroop congruent condition, while Stroop Incongruent means that the interaction of two effects was in the Stroop incongruent condition. Only significant and marginally significant results are reported.

In terms of the ERs, a main effect of the SNARC effect was observed, with *F*(1, 31) = 40.14, *p* < 0.001, *η_p_*^2^ = 0.56, and a larger ER in the SNARC incongruent condition (7.0 ± 0.8%) than in the SNARC congruent condition (3.0 ± 0.4%). A main effect of the cognitive Simon effect was observed, with *F*(1, 31) = 4.18, *p* = 0.050, *η_p_*^2^ = 0.12, and a larger ER in the Simon incongruent condition (5.6 ± 0.7%) than in the Simon congruent condition (4.4 ± 0.6%). The interaction between the cognitive Simon effect and the magnitude Stroop effect was not observed, *F*(1, 31) = 1.80, *p* = 0.189, *η_p_*^2^ = 0.06, BF_10_ = 0.47. No other significant main effects or interactions were observed, with all *p* values greater than 0.05.

In addition, the Pearson correlation coefficient of RT and ER was significant, with *r* = 0.200 and *p* = 0.001. Although there was no trade-off between the two, a significant positive correlation was observed. Therefore, the IES could be introduced for further ANOVAs.

In terms of the IESs, a main effect of the SNARC effect was observed, with *F*(1, 31) = 51.50, *p* < 0.001, *η_p_*^2^ = 0.62, and a greater IES in the SNARC incongruent condition (622 ± 13 ms) than in the SNARC congruent condition (563 ± 10 ms). A main effect of the cognitive Simon effect was observed, with *F*(1, 31) = 4.99, *p* = 0.033, *η_p_*^2^ = 0.14, and a greater IES in the Simon incongruent condition (599 ± 11 ms) than in the Simon congruent condition (586 ± 12 ms). The interaction between the cognitive Simon effect and the magnitude Stroop effect was not observed, *F*(1, 31) = 2.25, *p* = 0.144, *η_p_*^2^ = 0.07, BF_10_ = 0.39. No other significant main effects or interactions were observed, with all *p* values greater than 0.05.

#### Discussion

The null interaction between the cognitive Simon effect and the magnitude Stroop effect confirmed the independence of these two effects (Li et al., 2014). Importantly, an unexpected result is a three-way interaction among the SNARC, cognitive Simon, and magnitude Stroop effects for the RT, suggesting that these factors interacted with each other (this will be discussed further below). These findings indicated that the SNARC effect was affected by the magnitude-relevant interference information when the magnitude information was task-irrelevant. Combined with the results of Yan et al. (2021) (additive combination of the SNARC and parity Stroop effects), we could conclude that the magnitude relevance of the semantic representation stage-related interference information could also modulate the SNARC effect in the case that magnitude information is task-irrelevant. These results also provide evidence to support the claim that the magnitude relevance of the interference information is the primary factor influencing the SNARC effect.

### Experiment 2b

Experiment 2b was simplified from Experiment 2a, similar to Experiments 1a and 1b, to identify whether the result of Experiment 2a was reproducible when the target number range was reduced to four numbers (1, 2, 7, 8).

#### Participants

Thirty undergraduate students (18–21 years old, average age: 19.2 years old; 9 men) from Guangzhou University participated in Experiment 2b. All the participants reported that they had no history of neurological or psychiatric issues; all participants were right-handed and had normal or corrected-to-normal vision.

#### Apparatus, stimuli, design, and procedure

Experiment 2b was identical to Experiment 2a, except that the target number range was changed to four numbers (1, 2, 7, and 8).

#### Statistical analysis

The analysis procedure of Experiment 2b was the same as that of Experiments 1a, 1b, and 2a. The data of all participants were used for analysis, with a mean accuracy of 96.6%. The trials with errors, RTs shorter than 200 ms, or RTs greater than 3 standard deviations (7.9%) in each condition were excluded from the RT analysis.

#### Results

In terms of the RTs, a main effect of the SNARC effect was observed, with *F*(1, 29) = 39.29, *p* < 0.001, *η_p_*^2^ = 0.58, and a longer RT in the SNARC incongruent condition (566 ± 9 ms) than in the SNARC congruent condition (525 ± 8 ms). A main effect of magnitude Stroop effect was observed, with *F*(1, 29) = 6.38, *p* = 0.017, *η_p_*^2^ = 0.18, and a longer RT in the magnitude Stroop incongruent condition (548 ± 8 ms) than in the magnitude Stroop congruent condition (542 ± 8 ms). An interaction between the SNARC and cognitive Simon effects was observed, with *F*(1, 29) = 9.94, *p* = 0.004, and *η_p_*^2^ = 0.26. A simple effect analysis showed that the effect size of the SNARC effect in the Simon congruent condition (50 ± 7 ms) was larger than that in the Simon incongruent condition (31 ± 7 ms), with *t*(30) = 3.15, *p* = 0.004, and *d* = 0.58. Besides, the interaction between the cognitive Simon effect and the magnitude Stroop effect was not observed, *F*(1, 29) = 0.07, *p* = 0.800, *η_p_*^2^ < 0.01, BF_10_ = 0.19. No other significant main effects or interactions were observed, with all *p* values greater than 0.05.

**Figure 5:**
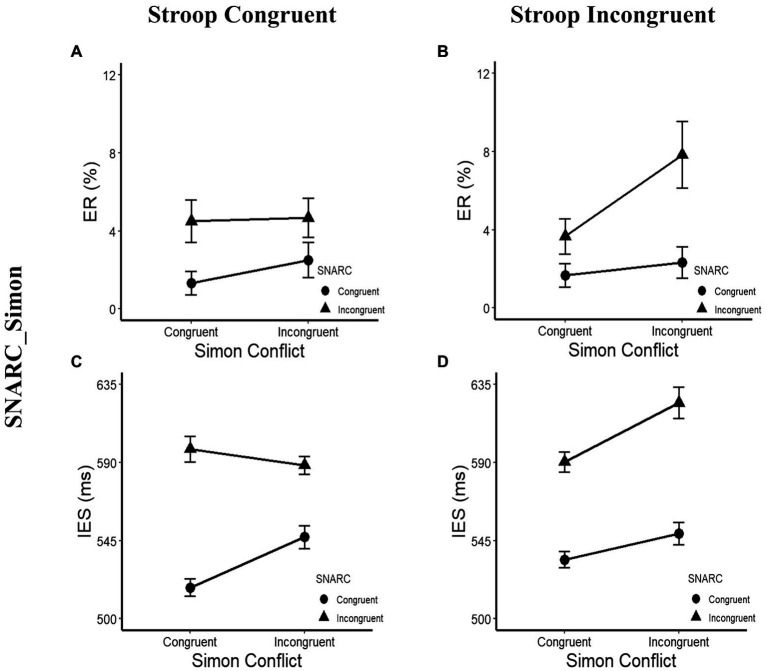
ERs and IESs in the *post hoc* multiple comparison of Experiment 2b. **(A,C)** Present the interaction between the SNARC and Simon effects in the Stroop congruent condition. The circle symbol represents the SNARC congruent condition, the triangle symbol represents the SNARC incongruent condition. **(B,D)** illustrate the interaction between the SNARC and cognitive Simon effects in the Stroop incongruent condition. The representation of circle and triangle symbols is the same as the **(A,C)**. The error bar represents the standard error.

In terms of the ERs, a main effect of the SNARC effect was observed, with *F*(1, 29) = 12.81, *p* = 0.001, *η_p_*^2^ = 0.31, and a larger ER in the SNARC incongruent condition (5.2 ± 0.8%) than in the SNARC congruent condition (2.0 ± 0.5%). Furthermore, a three-way interaction among the SNARC, magnitude Stroop and cognitive Simon effects was observed, with *F*(1, 29) = 4.28, *p* = 0.048, and *η*_p_^2^ = 0.13. A *post hoc* multiple comparison test showed that in the magnitude Stroop congruent condition, there was no interaction between the SNARC and cognitive Simon effects, with *F*(1, 29) = 0.54, *p* = 0.470, and *η_p_*^2^ = 0.02; however, in the magnitude Stroop incongruent condition, there was a marginally significant interaction between the SNARC and cognitive Simon effects, with *F*(1, 29) = 3.73, *p* = 0.063, and *η_p_*^2^ = 0.11, showing that the effect size of the SNARC effect in the Simon incongruent condition (5.5 ± 1.7%) tended to be larger than that in the Simon congruent condition (2.0 ± 1.1%; Figures 5A,B; Table 4). Besides, the interaction between the cognitive Simon effect and the magnitude Stroop effect was not observed, *F*(1, 29) = 0.03, *p* = 0.861, *η_p_*^2^ < 0.01, BF_10_ = 0.35. No other significant main effects or interactions were observed, with all *p* values greater than 0.05.

**Table 4:** *Post hoc* multiple comparison results of Experiment 2b for ERs and IESs.

			C/C	C/I	I/C	I/I
SNARC/Simon	Stroop congruent	ER (%)	1.3 (0.6)	2.5 (0.9)	4.5 (1.1)	4.7 (1.0)
		IES (ms)	518 (10)	547 (13)	598 (15)	588 (10)
	Stroop incongruent	ER (%)	1.7 (0.6)	2.3 (0.8)	2.7 (0.9)	7.8 (1.7)
		IES (ms)	534 (10)	549 (13)	590 (12)	624 (18)

C/C means that the SNARC effect and the Simon effect are both in the congruent condition, while I/C means that the SNARC effect is in the incongruent condition and the Simon effect is in the congruent condition. Stroop Congruent means that the interaction of two effects was in Stroop congruent condition, while Stroop Incongruent means that the interaction of two effects was in the Stroop incongruent condition. Only significant and marginally significant results are reported.

In addition, the Pearson correlation coefficient of RT and ER was significant, with *r* = 0.216 and *p* = 0.001, showing that there was a positive correlation but no trade-off between the two. Therefore, the IES could be introduced as a factor for further ANOVAs.

In terms of the IESs, a main effect of the SNARC effect was observed, with *F*(1, 29) = 32.53, *p* < 0.001, *η_p_*^2^ = 0.53, and a greater IES in the SNARC incongruent condition (600 ± 11 ms) than in the SNARC congruent condition (537 ± 10 ms). A main effect of the magnitude Stroop effect was observed, with *F*(1, 29) = 5.25, *p* = 0.029, *η_p_*^2^ = 0.15, and a greater IES in the Stroop incongruent condition (574 ± 9 ms) than in the Stroop congruent condition (563 ± 9 ms). A three-way interaction among the SNARC, magnitude Stroop and cognitive Simon effects was observed, with *F*(1, 29) = 10.74, *p* = 0.003, and *η_p_*^2^ = 0.27. A *post hoc* multiple comparison test showed that in the magnitude Stroop congruent condition, there was a significant interaction between the SNARC and cognitive Simon effects, with *F*(1, 29) = 9.49, *p* = 0.005, and *η_p_*^2^ = 0.25, showing that the effect size of the SNARC effect in the Simon congruent condition (80 ± 13 ms) was larger than that in the Simon incongruent condition (41 ± 14 ms), while in the magnitude Stroop incongruent condition, there was no interaction between the SNARC and cognitive Simon effects, with *F*(1, 29) = 0.91, *p* = 0.349, and *η_p_*^2^ = 0.03 (Figures 5C,D; Table 4). Besides, the interaction between the cognitive Simon effect and the magnitude Stroop effect was not observed, *F*(1, 29) = 0.05, *p* = 0.830，*η*_p_^2^ < 0.01，BF_10_ = 0.28. No other significant main effect or interactions were found, with all *p* values greater than 0.05.

#### Discussion

In Experiment 2b, an interaction between the SNARC and cognitive Simon effects was found in the RT, indicating that the SNARC effect is stably affected by the response-selection stage-related interference information. Similarly, the three-way interaction among the SNARC, cognitive Simon, and magnitude Stroop effects was observed, repeating the results of Experiment 2a. These findings confirmed that the SNARC effect is affected by magnitude-relevant interference information when the magnitude information is task-irrelevant, implying that the magnitude relevance of the interference information is the primary factor.

## General discussion

In this study, based on the experimental paradigm of Yan et al. (2021) and Nan et al. (2022), the semantic representation-related interference information (magnitude Stroop effect and parity Stroop effect) was switched between two tasks (magnitude comparison task and parity judgment task) to determine whether the magnitude relevance of interference information or task relevance of magnitude information influenced the SNARC effect. In Experiments 1a and 1b, a parity Stroop effect was induced in a magnitude comparison task; the results showed that the SNARC effect interacted with the cognitive Simon effect but not the parity Stroop effect. In Experiments 2a and 2b, a magnitude Stroop effect was induced in a parity judgment task; the results showed that the SNARC effect interacted with both the cognitive Simon and magnitude Stroop effects. Based on the interaction between the SNARC and magnitude Stroop effects in the magnitude comparison task of Nan et al. (2022) and the absence of interaction between the SNARC and parity Stroop effects in the parity judgment task of Yan et al. (2021), we concluded that the magnitude relevance of interference information might be the primary factor that influences the SNARC effect and that led to the previous contradictory results.

### The null interaction between the SNARC and parity Stroop effects in the magnitude comparison task

Numbers contain both magnitude information and parity information. A large number of studies on the SNARC effect have shown that the magnitude information is essential for the SNARC effect to be generated, while the parity information is not (Shaki and Fischer, 2018; Pinto et al., 2019b; Prpic et al., 2021). Namely, if the numerical magnitude representation is affected (e.g., the magnitude Stroop effect), the SNARC effect would be influenced. The factors influencing the numerical magnitude representation could come from the task type or the interference information during the task execution. Our results could reject the influence from the task type. Nan et al. (2022) found that the SNARC effect was affected by the magnitude Stroop effects in the magnitude comparison task, but we found that the SNARC effect was not affected by the parity Stroop effect under the same task (Experiment 1). These results suggested that the SNARC effect is affected when the semantic representation stage-related interference information has an impact on the numerical magnitude representation (the results of Experiment 2 further confirmed this finding).

However, it has also been suggested that the numerical ordinal sequences play an important role in actuating the SNARC effect (van Dijck and Fias, 2011; Casasanto and Pitt, 2019; Pitt and Casasanto, 2020). In typical digital tasks, it is difficult to accurately assess the involvement of the magnitude and ordinal sequences in the SNARC effect, because digits show both properties of magnitude and ordinal sequences at the same time (Prpic et al., 2016, 2021). Notably, the model proposed by Prpic et al. (2016) explains both the order and magnitude properties of the SNARC effect. Prpic et al. (2016) deemed that the SNARC effect possesses separate mechanisms for magnitude and order processing, which are spatially organized depending on task demands. In the task in which magnitude information is task-relevant, the order-related mechanism is preferentially activated and plays a predominant role; while in the task in which magnitude information is task-irrelevant, the magnitude-related mechanism is dominant. Our results do not deny this view. But we emphasized that the numerical information might be as dominant as the ordinal sequences in the task that magnitude information is task-relevant. The interaction between the SNARC and magnitude Stroop effects in the results of Nan et al. (2022) indicated that the numerical magnitude information was sufficiently semantic processed and thus affected the magnitude representation of the SNARC effect.

In addition, an interesting phenomenon is worth mentioning: the main effect of the parity Stroop effect was not observed in the magnitude comparison task, while the magnitude Stroop effect was observed in the parity judgment task. One possible explanation for this observation is that the magnitude information of the word has been overlearned. Thus, the magnitude information tends to be processed automatically, which affects number processing, while the parity information of the word is unfamiliar and thus does not produce an interference effect (Moors and De Houwer, 2006; Cleland and Bull, 2019).

### The three-way interaction among the SNARC, cognitive Simon, and magnitude Stroop effects in the parity judgment task

The results of the magnitude comparison task (Experiment 1) suggested that the magnitude relevance of the semantic representation stage-related interference information is the primary factor affecting the SNARC effect. The results of the parity judgment task (Experiment 2) could rule out the impact from the task relevance of magnitude information, and further confirmed the dominant influence of the magnitude relevance of interference information on the SNARC effect. The magnitude Stroop effect as interference information could affect the magnitude representation of the SNARC effect. Thus, Yan et al. (2021) found that the SNARC effect was not affected by the parity Stroop effect, while we observed that the SNARC effect was affected by the magnitude Stroop effect. These findings also implied that the SNARC effect involved the semantic representation stage.

Unexpectedly, there was a three-way interaction among the SNARC, magnitude Stroop and cognitive Simon effects. The Stroop and Simon effects have been shown to activate different cognitive processes (semantic-representation stage and response-selection stage); they are independent of one another and do not affect each other (Liu et al., 2010; Li et al., 2014; Scerrati et al., 2017). The null interaction between the cognitive Simon effect and the Stroop effect was indeed observed in Experiments 1 and 2. One plausible explanation is that the three effects were influenced by the involvement of the SNARC effect. Specifically, the magnitude information is related to the magnitude Stroop effect and the SNARC effect, while the spatial information is related to the cognitive Simon effect and the SNARC effect (Shaki and Fischer, 2018; Pinto et al., 2019b, 2021b). The magnitude and spatial information are both task-irrelevant in the parity judgment task; individuals inhibit the task-irrelevant information while enhancing the task-relevant parity information to ensure the correct response (Fox et al., 2001; Fenske and Eastwood, 2003), which might result in a competition among cognitive resources to inhibit task-irrelevant information, resulting in an interaction among these three effects. Therefore, this three-way interaction provided evidence that the SNARC effect was indeed affected by the magnitude Stroop effect, supporting the view that the magnitude relevance of interference information is the key factor to the SNARC effect.

### A two-stage processing model to explain the flexibility of the SNARC effect

**Figure 6:**
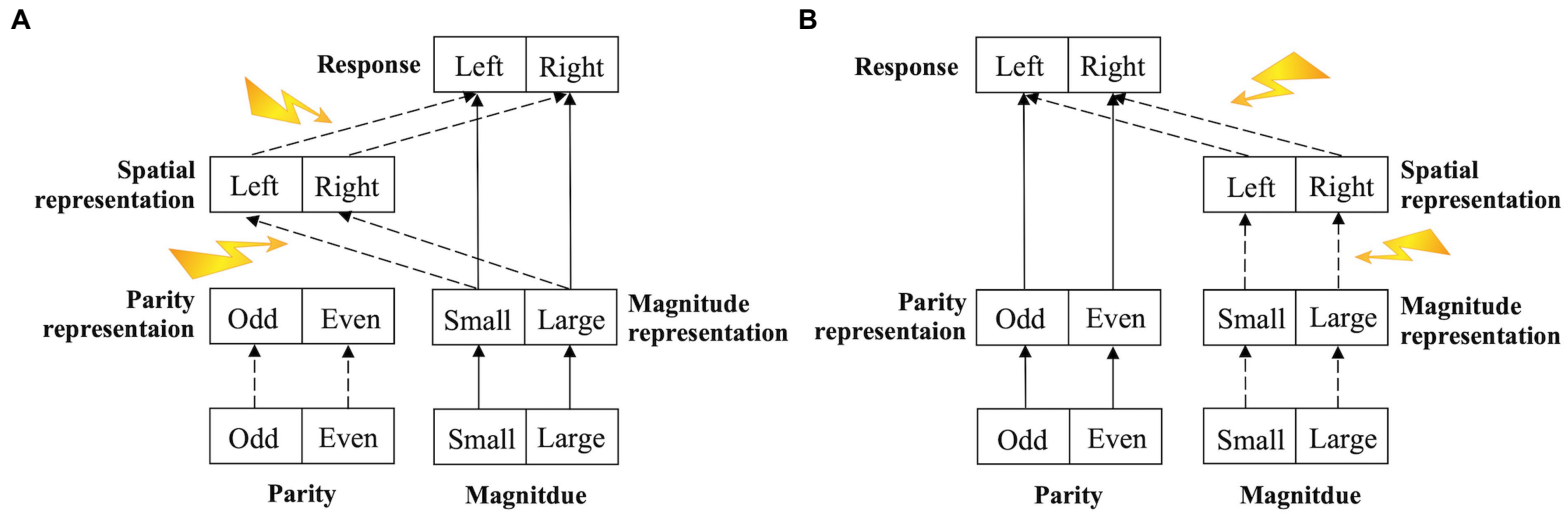
Structure of the two-stage processing model of the SNARC effect. The solid line indicates the pathway of the task-relevant (explicit) information, the dotted line indicates the pathway of the task-irrelevant (implicit) information, and the yellow arrow indicates various interference factors that act on the pathway of the SNARC effect. **(A)** The magnitude comparison task. The magnitude information is task-relevant (explicit) input, while the parity information is task-irrelevant (implicit) input. **(B)** The parity judgment task. The parity information is task-relevant (explicit) input, while the magnitude information is task-irrelevant (implicit) input.

Previous studies have shown that the SNARC effect is flexible in terms of the direction (left to right or right to left; Dehaene et al., 1993; Bächtold et al., 1998; Fias and van Dijck, 2016; Zhang et al., 2020) and the processing stage at which it occurs (the early semantic-representation stage, late response-selection stage, or both stages; Fischer et al., 2004; Daar and Pratt, 2008; Tlauka, 2010; Basso Moro et al., 2018; Pinto et al., 2021a). On the basis of our results and of previous literature, we proposed a two-stage processing model of the SNARC effect (a stage with the spatial representation of the magnitude and a stage with the spatial representation of the response selection, which are similar to the semantic-representation stage and response-selection stage, respectively, discussed in previous studies) to explain the flexibility of the SNARC effect (Figure 6). If any factor (e.g., reading habits, negative number, or numerical arrangement) acted on these two stages (Bächtold et al., 1998; Fischer et al., 2010; Han et al., 2017), the SNARC effect and its effect size would be affected, resulting in the various forms of the SNARC effect observed in previous studies.

The model included three levels (input, hidden, and output). In the input level, there were two layers. Each layer independently encoded a single piece of stimulus information. During a simple digital task (the magnitude comparison task or the parity judgment task), one layer encoded the numerical magnitude information (small or large), while the other layer encoded the numerical parity information (odd or even). In the hidden level, there were three layers. One layer, referred to as the magnitude representation layer, represented and processed the numerical magnitude information. Another layer, referred to as the parity representation layer, represented and processed the numerical parity information. These two layers are collectively known as the semantic-representation layer. The third layer, referred to as the spatial representation layer, represented and processed the spatial representation (left or right) that was automatically generated by the magnitude representation. In the output level, one layer received information from the semantic-representation layer and the spatial representation layer and encoded the response (left or right); this layer was referred to as the response layer. If the information in the semantic-representation layer and spatial representation layer conflicted, the response of the semantic-representation layer was enhanced, while the response of the spatial representation layer was inhibited, resulting in a longer RT and demonstrating the SNARC effect. This model could distinguish between the different magnitude information processing pathways in the magnitude comparison task (task-relevant) and the parity judgment task (task-irrelevant). The flexible variation in the SNARC effect observed in most previous studies can be explained by this model.

First, the factors associated with the magnitude representation (e.g., reading habit, working memory load, and numerical activation degree) affected the stage with the spatial representation of the magnitude. (1) Reading habits in long-term memory could affect the spatial representation of the magnitude, resulting in participants who read from left to right having a left-to-right SNARC effect, while those who read from right to left have a right-to-left SNARC effect (Dehaene et al., 1993; Zebian, 2005; Shaki and Fischer, 2008; Shaki et al., 2009). (2) Situational experience, or rather any experience that spatializes numbers, could transform the direction of magnitude representation temporarily or permanently, resulting in the reversal or vanishing of the SNARC effect (Bächtold et al., 1998; Pitt and Casasanto, 2020; Mingolo et al., 2021). (3) In the case of high working memory load, the numerical magnitude information from the numerical representation could not be processed sufficiently by the limited cognitive resources, resulting in the dilution or even reversal of the SNARC effect (Herrera et al., 2008; van Dijck et al., 2009; van Dijck and Fias, 2011). (3) The degree of numerical activation could affect the magnitude representation, resulting in the SNARC effect being observed only when the stimulus was determined to be a number or was processed for a long enough time in a color judgment task (Cleland and Bull, 2019); moreover, the SNARC effect varied with the distance between the target number and the reference number (Basso Moro et al., 2018).^1^ (4) According to the results of our study, the magnitude Stroop effect influenced the magnitude representation and thus the spatial representation of the magnitude, while the parity Stroop effect did not, resulting in an interaction between the magnitude Stroop effect and SNARC effect and no interactions between the parity Stroop effect and SNARC effect.

Second, the factors associated with the response selection (e.g., the Simon effect and the switching response rule) affected the stage with the spatial representation of the response selection. (1) In the task of combining the SNARC and Simon effects, regardless of whether the visual Simon effect was induced by the position of the number or the cognitive Simon effect was induced by the mutual interference between the cognitive coding of the number location and the reaction location, the spatial position of the number affected its spatial representation and further interfered with this stage, resulting in the dilution of the SNARC effect (Gevers et al., 2005; Keus et al., 2005; Treccani et al., 2009; Gut et al., 2021). Therefore, an interaction between the cognitive Simon effect and the SNARC effect was consistently observed in our study. (2) The switching response rule could directly affect the response selection, showing the interaction between the SNARC effect and the switch cost (Basso Moro et al., 2018; Zhang et al., 2020).

### Limitations and future directions

In our study, the main effects of the SNARC, cognitive Simon, and Stroop effects were not consistently observed across all four experiments, which may have influenced the comparison of the three effects. Future studies could attempt to stably induce these three effects under the task-irrelevant condition to better evaluate their relationship. In addition, the conflict adaptation effect, which is another index for determining the relationship between different conflicts (Gratton et al., 1992; Egner, 2008; Yang et al., 2017), could be adopted in future research to further assess the overlapping conflict processing mechanism among these three effects.

Moreover, with the proposed two-stage processing model, diverse interference factors could be induced in the future to investigate their influence on the SNARC effect. Three possible research directions could be taken. First, in the stage of the spatial representation of the magnitude, the load intensity of the input information could be changed to improve or reduce the cognitive load of the magnitude representation and processing (e.g., adding color information as a stimulus to improve the magnitude representation load) to examine its influence on the SNARC effect (Schuller et al., 2014; Deng et al., 2017; Wang et al., 2020). Second, in the stage of the spatial representation of the response selection, the set of responses could be changed (Schneider and Logan, 2014; Pinto et al., 2019a), such as expanding the responses from two buttons to four buttons to investigate its influence on the SNARC effect. Third, different observable indicators could be used to repeatedly test the current conclusions and determine the internal mechanism of the SNARC effect (Weis et al., 2015; Nikolaev et al., 2020). For example, the event-related potential (ERP) has a high temporal resolution (Keus et al., 2005), and functional magnetic resonance imaging (fMRI) has a high spatial resolution (Gut et al., 2021).

## Conclusion

The present findings showed that the SNARC effect was interactive with the cognitive Simon effect and additive with the parity Stroop effect in the magnitude comparison task (Experiments 1a and 1b), while it was interactive with both the cognitive Simon and magnitude Stroop effects in the parity judgment task (Experiments 2a and 2b). When combined with the results of Yan et al. (2021) and Nan et al. (2022), these findings indicated that the magnitude relevance of interference information related to the semantic-representation stage was the primary factor affecting the SNARC effect and supported the view that the SNARC effect occurs in both the semantic-representation stage and the response-selection stage. A new two-stage processing model of the SNARC effect (the stage of the spatial representation of the magnitude and the stage of the spatial representation of the response selection) was proposed to explain the flexibility of the SNARC effect. According to this model, different manipulation factors interfere with the two stages of the SNARC effect from different aspects, so there was flexible variation of the SNARC effect in previous studies.

## Data availability statement

The datasets presented in this study can be found in online repositories. The names of the repository/repositories and accession number (s) can be found below: All data, stimulus materials and code for experiment and data analysis have been made publicly available *via* the Open Science Framework, https://osf.io/q2yc7/.

## Ethics statement

The studies involving human participants were reviewed and approved by The Institutional Review Board of the Educational School, Guangzhou University (No. GZHU2020016). The patients/participants provided their written informed consent to participate in this study.

## Author contributions

WN, LY, and SF designed the study. XX and LY created the stimuli and designed the experiment. WN and XX oversaw the data collection and analyzed the data. WN, XX, LY, and SF wrote the manuscript. All authors contributed to the article and approved the submitted version.

## Funding

This research was supported by the Natural National Science Foundation of China (31970993) to SF, and the Youth Project of Basic and Applied Basic Research Fund of Guangdong Province – Regional Joint Fund (No. 2021A1515110452) to WN.

## Conflict of interest

The authors declare that the research was conducted in the absence of any commercial or financial relationships that could be construed as a potential conflict of interest.

## Publisher’s note

All claims expressed in this article are solely those of the authors and do not necessarily represent those of their affiliated organizations, or those of the publisher, the editors and the reviewers. Any product that may be evaluated in this article, or claim that may be made by its manufacturer, is not guaranteed or endorsed by the publisher.

## Footnotes

^1^In Experiments 1a and 2a (target number: 0, 1, 2, 3, 6, 7, 8, or 9), the numerical distance effect was analyzed to verify one aspect of the new two-stage processing model of the SNARC effect. A repeated-measures analysis of variance (ANOVA), with 2 × 2 × 2 × 2 factors used for the SNARC effect, Stroop effect, cognitive Simon effect and distance (close [2, 3, 6, and 7], far [0, 1, 8, and 9]), was conducted on the RTs. The results showed the main effect of the numerical distance. Moreover, the interaction between the SNARC effect and the distance was observed in both experiments (Experiment 1a: *F*(1, 31) = 6.20, *p* = 0.018, *η*_p_^2^ = 0.167; Experiment 2a: *F*(1, 31) = 10.33, *p* = 0.003, *η*_p_^2^ = 0.250), indicating that the numerical distance effect did influence the SNARC effect by affecting the spatial representation of the magnitude stage, supporting our two-stage processing model.
